# Authors' Reply

**DOI:** 10.1371/journal.pmed.0030288

**Published:** 2006-06-27

**Authors:** Jocelyn E Mackie, Andrew D Taylor, David L Finegold, Abdallah S Daar, Peter A Singer

**Affiliations:** **1**University of TorontoJoint Centre for Bioethics, Toronto, OntarioCanada; **2**Keck Graduate Institute of Applied Life SciencesClaremont, CaliforniaUnited States of America; **3**University of TorontoJoint Centre for Bioethics, Toronto, OntarioCanada

We thank Boyd and Rogers for their comments [ 1] about our article “Lessons on Ethical Decision Making from the Bioscience Industry” [ 2].

In response to their first point, we would like to refer them to our book, entitled *BioIndustry Ethics*, on which our article was based [ 3]. In the introduction of the book, we provide a discussion about the intersection of traditional medical ethics and business ethics as well as business strategy and explain that it is this intersection that is the focus of our study. The decisions made by management in bioscience companies (about what drugs to develop, where and how to conduct clinical trials, etc.) are not merely procedural but a combination of procedural and substantive ethical decision-making. We would also like to point out that the purpose of our article was to highlight the *mechanisms* being used by bioscience companies. It is our belief that by instituting processes that encourage ethical discussions (through an ethics department, an ethics advisory boards, with ethics education and forums for ethics discussion), management in bioscience companies will begin to make tough ethical decisions openly and consciously. We would like to reiterate that our primary audience for this article are decision-makers in bioscience companies who, as a first step, can learn from such approaches.

With respect to Boyd and Rogers' second point, as we write in the funding section of the paper, the primary funding for this study came from public sources. Moreover, as we also emphasise in our book, members of our research team with ties to a particular company did not participate in the case study of that company. Beyond our disclosing funding sources—which is, of course, necessary and appropriate—it is not clear what Boyd and Rogers are really asking for. They seem to feel our article is incomplete because it is missing an exegesis on how this funding might influence our results. In fact, we think they are trying to imply that it is inappropriate for anyone with industry funding to study ethical practices in industry. This is of course not the standard in clinical research: the standard is disclosure. But there is something more fundamental here. Our study is the very first, to our knowledge, to systematically document practices in bioscience companies with respect to ethical challenges. In clinical ethics, it took several decades after the advent of ethics committees for analogous studies to be conducted. Science has a simple solution to Boyd and Rogers' complaint—it's called replication. Boyd and Rogers themselves, or others, should roll up their sleeves and study industry practices rather than, like some bioethicists, simply cast aspersions about “intellectual honesty and self-critique.” But the logical extension of Boyd and Rogers' critique is that people within companies—or with real-world experience of companies' attempts to address ethical challenges—would be ineligible to conduct studies aimed at improving companies' practices in response to ethical challenges. This is the type of Alice in Wonderland world that is the end result of Boyd and Rogers' world view.
